# Volunteering in adolescence and young adulthood crime involvement: a longitudinal analysis from the add health study

**DOI:** 10.1186/s40621-016-0091-6

**Published:** 2016-11-21

**Authors:** Shabbar I. Ranapurwala, Carri Casteel, Corinne Peek-Asa

**Affiliations:** 1 Injury Prevention Research Center, Department of Occupational and Environmental Health, College of Public Health, University of Iowa, Iowa City, IA USA; 2 Department of Epidemiology, Gillings School of Global Public Health, University of North Carolina, Chapel Hill, NC USA

**Keywords:** Volunteering, Delinquency, Crime involvement, Adolescents, Arrests, Convictions

## Abstract

**Background:**

Experiences in adolescence may have a lasting impact on adulthood. The objective of this study is to evaluate the association between adolescent (12–18 years of age) volunteerism with the incidence of illegal behaviors, arrests, and convictions in adulthood (>18 years of age).

**Methods:**

We conducted a retrospective cohort study using secondary data from the National Longitudinal Study of Adolescent to Adult Health. Students from grades 7–12 were recruited in 1994–1995 (*n* = 20,745), and then followed in 2001–2002 (*n* = 14,322) and in 2008–2009 (*n* = 12,288). In 2000–2001, participants were retrospectively asked about their volunteering experience from 12 to 18 years of age. Consequently, participants were divided into non-volunteers, self-volunteers, adult-required volunteers, and court-ordered volunteers. Groups were compared for rates of illegal behaviors, arrest, and convictions in adulthood (>18 years of age) using weighted generalized linear mixed negative binomial models while accounting for sampling design.

**Results:**

Relative to non-volunteers, self-volunteers reported 11 % fewer illegal behaviors (RR: 0.89, 95 % CI: 0.80, 0.99), 31 % fewer arrests (RR: 0.69, 95 %: 0.57, 0.85), and 39 % fewer convictions (RR: 0.61, 95 % CI: 0.47, 0.79) by age 18–28 years, and 28 % fewer illegal behaviors, 53 % fewer arrests, and 36 % fewer convictions by age 24–34. In comparison the adult-required volunteers also reported fewer arrests and convictions; however, they reported more illegal behaviors than the non-volunteers. The court-ordered volunteers reported higher rates of criminal involvement than the non-volunteers, throughout.

**Conclusion:**

This study suggests that volunteering in adolescence may reduce crime involvement in adulthood.

**Electronic supplementary material:**

The online version of this article (doi:10.1186/s40621-016-0091-6) contains supplementary material, which is available to authorized users.

## Background

Youth violence and crime is a core public health problem of the 21st century United States, especially in large urban areas (Federal Bureau of Investigation 2013). Studies show that arrest rates for all crimes increase sharply up to 20–25 years of age, and then decline (BJS 2014; Johnson et al. 2015; Moffitt 1993; Snyder, 2012). In 2010, the peak rate of arrests for murder, forcible rape, robbery, aggravated assault, and simple assault were between 16 and 21 years of age (Snyder, 2012). The median ages for these crimes were between 21 and 29 years age suggesting that half of the violent crimes were committed by individuals younger than 21 to 29 years of age. During the same time 9 % murders, 14 % rapes, 21 % robberies, 11 % aggravated assaults, and 16 % simple assaults involved adolescents or children (Snyder, 2012).

Experiences during adolescence have a great impact on the aspirations, conduct, health, and achievement during adulthood and throughout life. Hence, many youth violence prevention programs target this age group and focus on increasing pro-social behavior by improving self-regulation, self-control, conflict resolution, peer mediation, and other social skills sets (Herrenkohl et al. 2000; Buckner et al. 2003).

Studies have shown that volunteerism or community service also increases resilience, prosocial thinking and behavior, sense of community belonging, social responsibility, and overall level of happiness among youth (Batchelder and Root 1994; Giles and Eyler 1994; Reed et al. 2005), by enhancing sense of self-worth (Raskoff and Sundeen 1999). Volunteering or community service can be defined as voluntary unpaid activities aimed at helping others; some examples include visiting the elderly home, preparing food for the homeless, or serving at a soup kitchen (Raskoff and Sundeen 1999). Community service may be completely voluntary or required by school, parents, religious groups, or the court of law. Community service has been shown to reduce sexual risk taking behaviors among adolescents (O’Donnell et al. 2002) and the incidence of self-reported teenage pregnancy (Allen et al. 1994). However, only one youth violence prevention program includes community youth service as an intervention, as a result of which self-reported violent behaviors among urban adolescents over a 6 month period declined (O’Donnell et al. 1999). Other cross-sectional studies have examined the association of delinquency with community service in conjunction with school sports involvement and extracurricular activities, and found that community service is associated with fewer delinquent behaviors in adolescents (Hoffman and Xu 2002; Crean 2012). In addition, two recent longitudinal studies from Denmark suggest that convicts who received community service sentences as opposed to prison terms were less likely to be involved in violent crime (Andersen, 2015) or reconvicted (Klement 2015). However, there have been no long term follow up studies that address how volunteering during adolescence may affect illegal or criminal activity during adulthood.

In this study, the authors use data from a nationally representative longitudinal study of adolescents to evaluate the association of self-reported volunteering between 12 and 18 years of age with the incidence of self-reported illegal behaviors, arrests, and convictions in adulthood.

## Methods

**Fig. 1:**
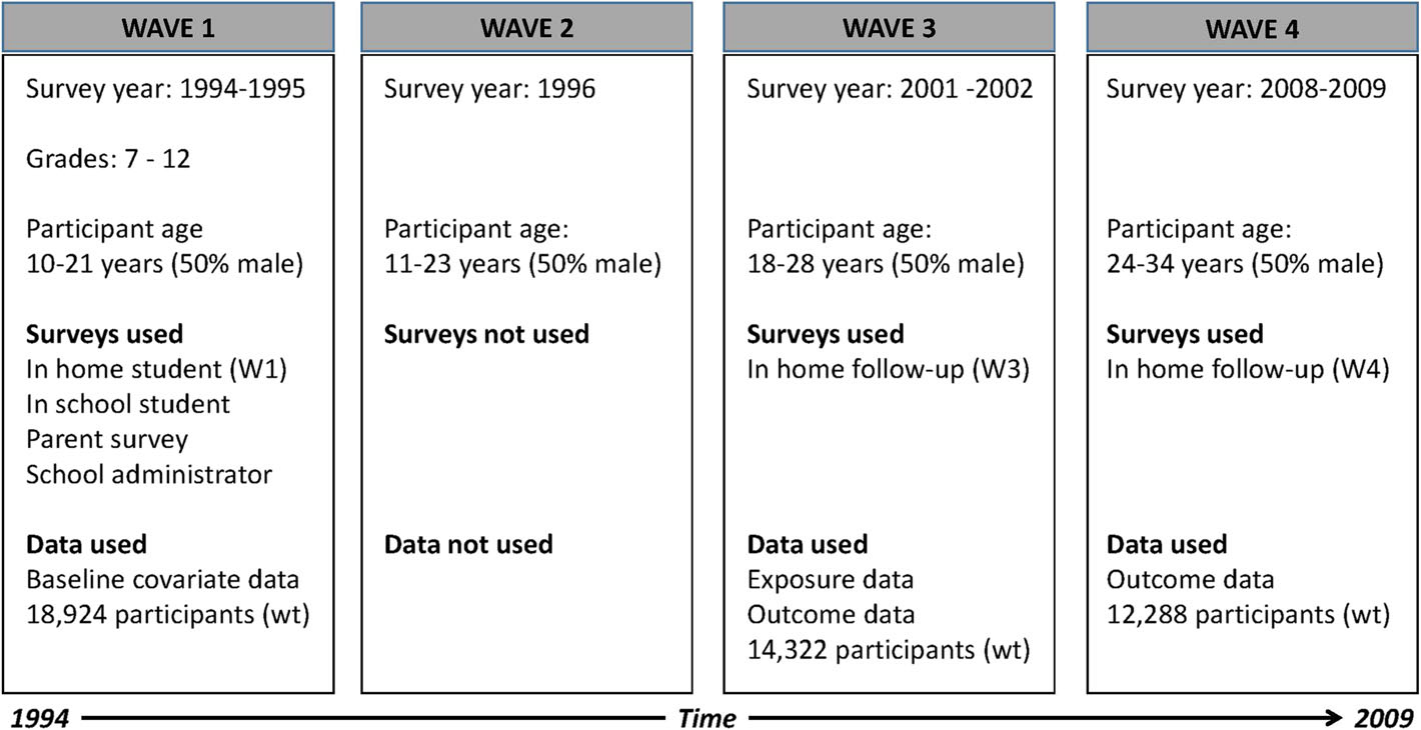
Schematic of Add Health data used for this study. *Abbreviations*: W1, wave 1; W3, wave 3; W4, wave 4; wt, weighted

A retrospective cohort study was conducted using data from the National Longitudinal Study of Adolescent to Adult Health (Add Health), where students were recruited from grades 7 through 12 in 1994–1995 (wave 1), and were followed in three subsequent waves during 1996 (wave 2), 2001–2002 (wave 3), and 2008–2009 (wave 4). Participant age in wave 1 ranged between 10 and 21 years, and by wave 4, these participants were between 24 and 34 years old. For this study, we used data from self-reported surveys conducted during waves 1, 3, and 4, as shown in Fig. 1.

In wave 1, 18,924 participants took the in-home surveys, 14,322 participants were then followed up in wave 3, of whom 12,288 were followed up in wave 4. The initial recruitment was carried out from a sample of 80 high schools and 52 middle schools in the United States with unequal selection probabilities stratified within four regions of the United States—West, Midwest, South, and the Northeast. The systematic sampling method allowed generalizability to all the schools in the United States with respect to the region of the country, urbanicity, size, type of the school (public, private, and Catholic), and ethnic distributions within the schools. More information about Add Health survey design can be found on the Carolina Population Center website [http://www.cpc.unc.edu/projects/addhealth], or in the description provided by Harris (Harris 2013). The current retrospective cohort study was approved by the University of Iowa Institutional Review Board.

### Outcomes

The main outcome variables were the rates of illegal behaviors, arrests, and convictions. The data on illegal behaviors, arrests, and convictions were collected in both waves 3 and 4.

Illegal behaviors: The participants were asked 13 questions in wave 3 (12 in wave 4) about their illegal behaviors during the previous 12 months. These questions pertained to intentional property damage; stealing more than $50; entering a house to steal something; threatening to use a weapon; selling marijuana or drugs; stealing something less than $50; being in a group physical fight; buying, selling or holding stolen property; doing credit card fraud; deliberately writing a bad check; using a weapon in a fight; carrying a gun to school or work, and; belonging to a named gang. The survey questionnaires can be accessed on the Carolina Population Center’s website (http://www.cpc.unc.edu/projects/addhealth/documentation). Participants reported the number of times (zero / 1 or 2 times / 3 or 4 times / 5 or more times) they had been involved in the above mentioned illegal behaviors. The responses were coded as zero = 0, 1 or 2 times =1, 3 or 4 times = 3, and 5 or more times = 5. These were then summed across all the questions to get a total count for the number of illegal behaviors during the past 12 months.

Arrests: Participants reported arrests since age 18 as absolute counts in both waves 3 and 4. We used these unaltered counts for arrest outcomes in waves 3 and 4.

Convictions: In wave 3, convictions since age 18 were reported as absolute counts. We used these unchanged counts for wave 3 conviction outcomes. However, in wave 4, convictions were reported as an ordinal variable with values ‘none,’ ‘once,’ and ‘more than once.’ To measure the rate of convictions from wave 4, we converted this ordinal variable into a count variable, such that none = 0, once = 1, and more than once = 2 if those reporting ‘more than once’ reported two or fewer convictions in wave 3. However, if a participant reported to have been convicted ‘more than once’ during the wave 4 survey, but had reported a conviction count of greater than ‘2’ in wave 3, then their conviction count for wave 4 was kept the same as the number of convictions they reported in wave 3. The arrest and conviction rates involve arrests and convictions as a result of both violent and non-violent crimes.

### Exposure

The main exposure variable was volunteering, reported as a four category variable. In wave 3, participants were asked if they regularly participated in volunteer or community service work between the ages of 12 and 18 years, not counting car washing or selling candy to raise money. If they said yes, they were further asked if this work was strictly voluntary, ordered by a court, or required by parents, school, or religious group; they could have checked one or more of these. Based on the above two questions we created a four category volunteering exposure: 1) Those who said that they ‘did not volunteer’ between 12 and 18 years of age were considered non-volunteers. 2) Among the volunteers, all those who said that they were ‘ordered to volunteer by the court’ were put in the court-ordered volunteers group, even if they had checked the other groups. 3) From the remaining volunteers, all those who said that they volunteered because it was ‘required by parents, school, or religious groups’ were put in the adult-required volunteers group. 4) The volunteers who remained then were all those who exclusively said that their volunteering activities were strictly voluntary, hence they were put in self-volunteers group. Thus, the four category adolescent volunteering exposure was, non-volunteers, self-volunteers, adult-required volunteers, and court-ordered volunteers.

### Confounding factors

A directed acyclic graph (DAG) (Greenland et al. 1999) was developed using previously published literature to assess confounding of the exposure-outcome association (Herrenkohl et al. 2000; US DOJ 1999; McKinney 2002; Sariaslan et al. 2013; Erez et al. 2008; Lansford et al. 2014; Rew and Wong 2006; Gibson 2008). The DAG is provided as a supplemental file (Additional file 1). The DAG helps identify covariates, which when controlled for, can block or control confounding from all observed non-causal pathways (observed confounding), while keeping the causal pathways open (that is, not controlling for causal intermediates) (Greenland et al. 1999). From the DAG identified minimal sufficient set of variables, we identified a set of parsimonious variables. The parsimony was determined by using precision-validity trade-off such that any variable that reduces the bias (or increases validity) more than it increases the variance (or reduces precision) is included in the final model (Greenland et al. 2016). The final minimal sufficient set of parsimonious variables included in the regression models to control for all observed or known confounding were: participant age, sex (male/ female), and race (White/ African American/ Native American/ Asian) when outcomes were collected (i.e., wave 3 or wave 4), and baseline characteristics collected during the wave 1 interview of participants and parents. The baseline characteristics were perceived neighborhood safety (safe/unsafe); perceived relationships with parents (caring/ not caring), friends (caring/ not caring), and teachers (caring/ not caring); presence of a mentor figure (yes/ no); adolescent delinquency score (measured as a count, similar to the illegal behavior count, but from wave 1); religiosity during adolescence (not religious/ non-practicing/ religious); parent education (less than high school/ high school graduate/ some college/ college graduate +); parent civic engagement (engaged/ not-engaged); family’s use of food stamps as a measure of socioeconomic status (yes/ no) (Scharoun-Lee et al. 2009); school suspension (yes/ no); school expulsion (yes/ no); worked for pay (yes/ no); and received allowance (yes/ no). Religiosity was measured as a categorical variable coded as not religious, non-practicing, or religious, based on participants frequency of attending religious services and observing religious rituals. Parent’s civic service engagement was defined as being a member of a parent/ teacher organization, military veterans’ organization, labor union, hobby or sports group, civic or social organization, and if the parents fund-raised or volunteered for their child’s school during the school year they completed the wave 1 Add Health parent questionnaire. Neighborhood safety variable was developed based on a question asked of the participants in wave 1, “Do you usually feel safe in your neighborhood?” Those who responded ‘yes’ were considered to be living in safe neighborhoods. Variables on participants perception of relationship with parents, teachers, and friends were developed based on questions asked in wave 1, “How much do you think your parents care about you?” Participants who responded with ‘somewhat,’ ‘quite a bit,’ or ‘very much,’ were considered to be having caring parents; likewise for teachers and friends.

### Statistical analysis

We used wave 3 longitudinal weights to calculate weighted descriptive statistics for all covariates by exposure status, and reported their 95 % confidence intervals (CI). We calculated rates for illegal behaviors, arrests, and convictions, where the outcome counts were the numerator and the person-time during which the respective outcome occurred was the denominator. The person-time for each participant’s illegal behaviors in both waves 3 and 4 was 1 year, and the person-time for arrests and convictions was obtained by subtracting 18 from the participant’s age in the respective data collection wave.

Survey statistics were used to calculate weighted covariate distributions and their 95 % CI by volunteering status. Weighted generalized linear mixed models, with identity link, were used to calculate crude and adjusted rate differences and 95 % CI, and weighted generalized linear mixed models for negative binomial distribution were used to calculate crude and adjusted rate ratios (RR) of illegal behaviors, arrests, and convictions comparing those who volunteered (self, adult-required, or court-ordered) in adolescent years to non-volunteers. We included clustering by school (random intercept) and stratifying by region, as suggested by Chen and Chantala (Chen and Chantala 2014), to adequately account for the sampling design.

To account for the dropout from wave 3 to wave 4, we conducted inverse probability of attrition weighting (Weuve et al. 2012). Inverse probability weights for continuing in the study in wave 4 were calculated for all participants conditioned on their participation in wave 3. We created a binary variable for dropping out, D4, in wave 4. If the participant dropped out in wave 4 but participated in wave 3, then D4 = 1, and if they didn’t dropout then D4 = 0. The inverse probability of attrition weighting for each participant in wave 4 was given by:

$$ \mathrm{Stabilized}\ \mathrm{weights} = \mathrm{P}\left(\mathrm{D}4=0\right)\ /\ \mathrm{P}\left(\mathrm{D}4=0\Big|\mathrm{L}3,\ \mathrm{L}1\right) $$

Where, P represents probability; L3 represents the covariates at wave 3, namely, volunteering, sex, age, and race; and L1 represents all DAG identified baseline covariates from wave 1.

The probabilities of continuing were derived using logistic regression. The stabilized weights thus obtained (minimum = 0.9; maximum = 1.4; mean = 1) were then multiplied with the longitudinal grand sample weights for waves 1, 3, and 4 provided by the Carolina Population Center for the Add Health Study (GSWGT134). This conjoined weight variable was used to run crude and adjusted generalized linear mixed models (with identity link for rate differences, and negative binomial distribution for rate ratios) to compare adulthood outcomes among different adolescent volunteer groups. We also ran the crude and adjusted generalized linear mixed models with only GSWGT134 weights, without our own stabilized inverse probability weights.

We examined effect measure modification of the exposure–outcome relationship due to participant demographic factors by including interaction terms of exposure (volunteering) and participant age, sex, and race.

We conducted sensitivity analysis to examine the validity of our count measure of convictions from wave 4, which was originally reported as an ordinal variable. To do so we used a proportional odds regression analysis to model the original ranked wave-4 conviction variable with three responses: no convictions, one conviction, and more than one conviction. All analyses were conducted in SAS 9.4 (SAS institute, Cary, NC).

## Results

**Fig. 2:**
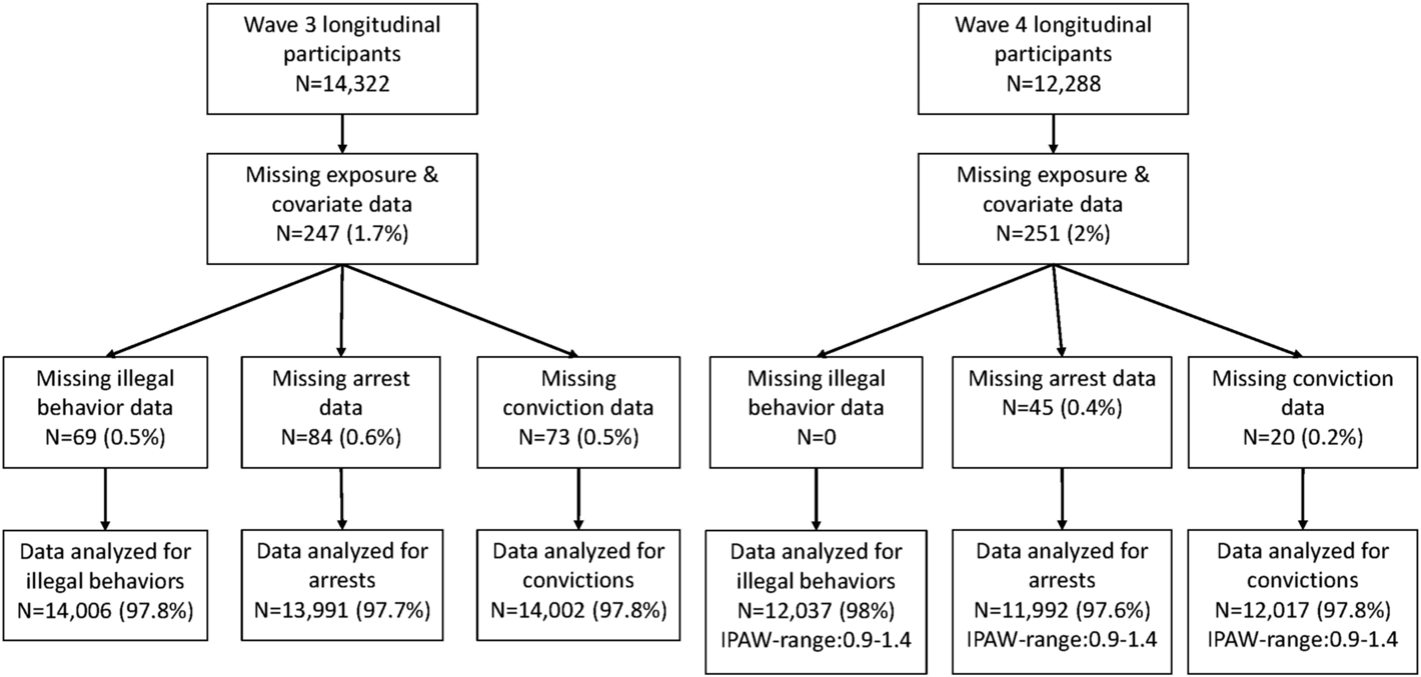
Available data for all outcome analyses during wave 3 and wave 4. *Abbreviations*: IPAW, inverse probability of attrition weights

Among the 14,322 participants followed in wave 3, 8003 (58 %) reported never volunteering during 12–18 years of age; 4695 (31 %) reported self-volunteering; 1238 (8 %) reported adult-required volunteering; 317 (2.4 %) reported court-ordered volunteering; and 69 participants had missing information. In wave 3, 247 participants (1.7 %) had missing exposure or covariate data, while in wave 4, 251 participants had missing covariate or exposure data. In addition less than 1 % of participants had missing outcome data in both waves. Participants with missing exposure, covariate, or outcome data were excluded from the analyses (Fig. 2).

Participant characteristics during wave 3 suggest that, participants who volunteered were the same age as the non-volunteers (Table 1). Participants who were required to volunteer by their adults (parents, religious groups, school), more often than not, believed their neighborhood was safe; believed that their parents and teachers cared for them; received allowance, and; had a mentor figure in life, as compared to other groups (Table 1). They were also most religious, had most educated parents, and their parents participated in civic activities more than the parents of participants from any other group (Table 1). The participants who were ordered by the court to volunteer had the highest delinquency score, were suspended and expelled, from school at baseline, the most among all other groups of participants, had the highest prevalence of food stamp use in their families, and the highest prevalence of ‘work-for-pay’ at baseline compared to other three groups. Participants who self-volunteered had the lowest mean delinquency score (Table 1).

**Table 1:** Distribution of wave 3 participant characteristics at wave 3 and baseline (wave 1) by adolescent volunteering

Participant characteristics during wave-3		Adolescent volunteering (weighted % or mean, and 95 % CI)^a^				
		None (*N* = 8003)	Self (*N* = 4695)	Adult required (*N* = 1238)	Court ordered (*N* = 317)	TOTAL (*N* = 14,322)
Mean age		22.5 (22.2, 22.7)	22.2 (22.0, 22.5)	21.9 (21.6, 22.2)	22.1 (21.8, 22.5)	22.4 (22.1, 22.6)
Male		53.2 (51.7, 54.7)	45.7 (43.7, 47.7)	42.7 (38.7, 46.7)	83.1 (77.7, 88.6)	50.8 (49.6, 52.0)
White^b^		75.5 (70.4, 80.7)	79.8 (75.3, 84.2)	71.4 (64.4, 78.5)	73.6 (65.0, 82.2)	76.4 (71.6, 81.2)
African American		18.0 (13.4, 22.6)	13.8 (10.2, 17.4)	19.0 (12.4, 25.6)	18.2 (11.0, 25.4)	16.8 (12.6, 20.9)
Native Americans		2.7 (1.4, 3.9)	2.3 (1.4, 3.2)	2.7 (1.5, 3.9)	3.5 (0.6, 6.4)	2.6 (1.6, 3.6)
Asian		3.8 (2.3, 5.4)	4.2 (2.3, 6.0)	6.8 (3.3, 10.4)	4.7 (−0.1, 9.4)	4.2 (2.5, 5.9)
Baseline characteristics (wave-1)						
Believed neighborhood was safe		87.7 (86.0, 89.4)	91.1 (89.4, 92.8)	93.0 (91.0, 94.9)	90.5 (86.6, 94.4)	89.2 (87.8, 90.7)
Believed parents care		94.5 (93.7, 95.3)	97.1 (96.5, 97.7)	97.6 (96.6, 98.6)	95.4 (92.8, 97.9)	95.6 (95.1, 96.1)
Believed friends care		81.7 (80.3, 83.2)	88.6 (87.1, 90.1)	87.7 (84.9, 90.5)	81.7 (74.1, 89.2)	84.4 (83.2, 85.6)
Believed teachers care		47.0 (44.8, 49.2)	59.1 (56.3, 62.0)	61.2 (56.8, 65.6)	38.4 (30.6, 46.1)	51.8 (49.8, 53.9)
Had a mentor figure^c^		70.1 (67.7, 72.5)	84.1 (82.6, 85.5)	85.7 (83.0, 88.5)	72.1 (66.2, 78.1)	75.8 (74.0, 77.6)
Mean delinquency score		4.4 (4.1, 4.6)	3.5 (3.3, 3.7)	3.7 (3.2, 4.1)	7.1 (6.0, 8.1)	4.1 (3.9, 4.3)
Religiosity	Non-religious	17.2 (15.3, 19.1)	10.4 (8.8, 12.0)	7.4 (5.4, 9.5)	16.1 (11.3, 20.8)	14.3 (12.7, 15.8)
	Non-practicing	13.6 (12.3, 14.8)	8.6 (7.4, 9.8)	6.1 (4.3, 7.9)	17.5 (12.1, 22.9)	11.5 (10.5, 12.5)
	Very religious	69.2 (66.8, 71.7)	81.0 (79.0, 83.1)	86.5 (83.3, 89.6)	66.4 (59.9, 72.9)	74.3 (72.2, 76.3)
Parent Education	Less than HS	57.3 (54.4, 60.2)	43.2 (39.6, 46.8)	40.3 (35.6, 44.9)	56.1 (48.0, 64.2)	51.4 (48.5, 54.2)
	HS graduate	28.5 (26.6, 30.4)	29.6 (27.2, 32.0)	28.7 (25.2, 32.1)	25.3 (19.0, 31.5)	28.9 (27.3, 30.5)
	Some College	9.6 (8.2, 10.6)	15.7 (13.5, 17.9)	17.9 (14.7, 21.0)	10.4 (5.0, 15.9)	12.1 (10.8, 13.3)
	College grad	4.8 (3.7, 5.9)	11.5 (9.1, 13.9)	13.2 (10.2, 16.7)	8.2 (4.7, 11.6)	7.7 (6.1, 9.2)
Parents engaged in civic services		43.0 (40.6, 45.4)	58.1 (54.9, 61.3)	58.2 (53.4, 62.9)	48.0 (40.0, 55.9)	49.2 (46.7, 51.7)
Food stamp participants		12.8 (10.5, 15.1)	6.7 (5.1, 8.3)	8.1 (5.3, 10.9)	14.3 (8.2, 20.4)	10.6 (8.6, 12.5)
Ever suspended from school		32.0 (29.1, 34.9)	18.7 (16.3, 21.1)	16.2 (12.9, 19.4)	53.1 (45.7, 60.4)	27.0 (24.4, 29.6)
Ever expelled from school		5.5 (4.5, 6.5)	2.2 (1.5, 2.9)	3.3 (1.4, 5.3)	10.7 (6.3, 15.1)	4.4 (3.5, 5.2)
Worked for pay		57.6 (54.6, 60.6)	63.0 (60.1, 66.0)	54.3 (49.3, 59.4)	65.3 (58.3, 72.3)	59.2 (56.5, 61.9)
Received allowance		43.7 (41.2, 46.3)	48.4 (45.6, 51.1)	51.6 (47.1, 56.1)	48.3 (41.1, 55.5)	45.9 (43.7, 48.2)

*HS* High school

^a^ missing adolescent volunteering information for 69 participants

^b^ missing race information for 206 participants

^c^ missing mentor information for 50 participants

The overall rate of illegal behaviors at wave 3 was 117 (95 % CI: 109, 126) per 100 person-years, while that in wave 4 was 54.1 (95 % CI: 48.0, 60.2) per 100 person-years. The overall rates of arrests and convictions at wave 3 were 5.0 (95 % CI: 4.3, 5.7) and 2.4 (95 % CI: 1.9, 2.8) per 100 person-years, respectively, while those in wave 4 were 5.2 (95 % CI: 4.4, 6.0) and 1.5 (95 % CI: 1.3, 1.7) per 100 person-years, respectively.

As compared to the non-volunteers, self-volunteers reported 11 % fewer illegal behaviors (RR: 0.89, 95 % CI: 0.80, 0.99), 31 % fewer arrests (RR: 0.69, 95 %: 0.57, 0.85), and 39 % fewer convictions (RR: 0.61, 95 % CI: 0.47, 0.79) by age 18–28 years (wave 3), and reported 28 % fewer illegal behaviors (RR: 0.72, 95 % CI: 0.59, 0.88), 53 % fewer arrests (RR: 0.47, 95 % CI: 0.38, 0.58), and 36 % fewer convictions (RR: 0.64, 95 % CI:0.51, 0.80) by age 24–34 (wave 4). Similarly, compared to non-volunteers, the adult-required volunteers reported 20 % more illegal behaviors, 37 % fewer arrests, and 29 % fewer convictions by wave 3, and 10 % more illegal behaviors, 29 % fewer arrests, and 19 % fewer convictions by wave 4. On the other hand those who were ordered to volunteer by court during their adolescence years reported higher illegal behaviors, arrests, and convictions than the non-volunteers by wave 3 and wave 4 (Table 2).

**Table 2:** Rates of illegal behaviors, arrests, and convictions by volunteering status, and unadjusted and adjusted rate differences and rate ratios of comparison

Outcomes Wave 3	Volunteering Type	Rate/ 100 PY (95 % CI)	Weighted^b^ rate differences (95 % CI)		Weighted^b^ rate Ratios (95 % CI)	
			Unadjusted	Adjusted^a^	Unadjusted	Adjusted^a^
Illegal behaviors	None (Referent)	120 (111, 130)	0	0	1	1
	Self	100 (89, 111)	-20 (-31, -9.9)	-7.7 (-18, 2.7)	0.83 (0.77, 0.88)	0.89 (0.80, 0.99)
	Adult-required	139 (121, 157)	19 (0.79, 36)	29 (11, 46)	1.1 (1.0, 1.3)	1.2 (1.0, 1.4)
	Court-Ordered	328 (298, 359)	208 (177, 238)	145 (115, 175)	2.8 (2.4, 3.3)	1.9 (1.5, 2.6)
Arrests	None (Referent)	5.4 (4.6, 6.1)	0	0	1	1
	Self	3.1 (2.2, 4.1)	-2.2 (-3.1, -1.4)	-1.0 (-1.9, -0.2)	0.59 (0.48, 0.74)	0.69 (0.57, 0.85)
	Adult-required	3.4 (1.9, 4.9)	-1.9 (-3.4, -0.48)	-0.8 (-2.2, 0.7)	0.61 (0.41, 0.91)	0.63 (0.44, 0.90)
	Court-Ordered	21 (19, 24)	16 (14, 19)	11 (8.9, 14)	5.1 (2.9, 8.7)	3.2 (2.1, 4.8)
Convictions	None (Referent)	2.7 (2.2, 3.2)	0	0	1	1
	Self	1.3 (0.7, 1.9)	-1.4 (-1.9, -0.88)	-0.97 (-1.5, -0.43)	0.49 (0.37, 0.65)	0.61 (0.47, 0.79)
	Adult-required	2.3 (1.4, 3.3)	-0.38 (-1.3, 0.53)	0.08 (-0.84, 1.0)	0.63 (0.39, 1.0)	0.71 (0.45, 1.1)
	Court-Ordered	9.6 (8.0, 11)	6.9 (5.3, 8.4)	4.4 (2.8, 6.0)	4.1 (2.2, 7.5)	2.9 (1.8, 4.8)
Wave 4						
Illegal behaviors	None (Referent)	61 (54, 68)	0	0	1	1
	Self	36 (28, 44)	-25 (-33, -18)	-14 (-21, -6.5)	0.61 (0.54, 0.70)	0.72 (0.59, 0.88)
	Adult-required	56 (44, 69)	-4.9 (-18, 7.8)	7.4 (-5.2, 19.9)	0.86 (0.69, 1.1)	1.1 (0.82, 1.5)
	Court-Ordered	147 (125, 169)	85 (64, 107)	53 (31, 74)	2.4 (1.7, 3.4)	1.5 (0.92, 2.5)
Arrests	None (Referent)	6.1 (5.2, 6.9)	0	0	1	1
	Self	2.3 (1.3, 3.2)	-3.8 (-4.6, -3.0)	-2.2 (-3.0, -1.4)	0.37 (0.32, 0.43)	0.47 (0.38, 0.58)
	Adult-required	2.9 (1.4, 4.3)	-3.2 (-4.6, -1.9)	-1.3 (-2.7, 0.03)	0.47 (0.36, 0.60)	0.71 (0.49, 1.0)
	Court-Ordered	15 (12, 17)	8.6 (6.2, 11)	4.8 (2.5, 7.1)	2.1 (1.4, 3.3)	2.1 (1.1, 3.9)
Convictions	None (Referent)	1.7 (1.5, 1.9)	0	0	1	1
	Self	0.89 (0.68, 1.1)	-0.79 (-0.97, -0.60)	-0.55 (-0.74 -0.36)	0.57 (0.50, 0.65)	0.64 (0.51, 0.80)
	Adult-required	1.1 (0.77, 1.4)	-0.57 (-0.89, -0.25)	-0.29 (-0.61, 0.03)	0.61 (0.49, 0.77)	0.81 (0.56, 1.2)
	Court-Ordered	4.1 (3.5, 4.6)	2.4 (1.8, 2.9)	1.7 (1.1, 2.2)	2.3 (1.8, 3.0)	2.0 (1.2, 3.3)

*PY* person-years

^a^ adjusted for age, sex, and race of the participant during respective survey waves, neighborhood safety, participant’s perception of relationships with parents, friends, and teachers, presence of a mentor figure in participant’s life, adolescent delinquency, adolescent religiosity, parent education, parent civic engagement, family’s use of food stamps, school suspension, school expulsion, work for pay, and allowance

^b^ the weighting for wave 3 is based on the survey weights, and the weighting for wave 4 is the product of survey weights and inverse probability of attrition weights

The adjusted rate differences suggest that for every 1000 participants followed-up every year (rate difference from Table 2 multiplied by 10), those who self-volunteered during adolescence (12–18 years of age) had 140 fewer illegal behaviors (95 % CI: -210, -65), 22 fewer arrests (95 % CI: -30, -14), and six (5.5) fewer convictions (95 % CI: -7.4, -3.6) by age 24–34 years of age (wave 4), relative to the non-volunteers (Table 2).

We did not observe any effect measure modification of the volunteering–crime involvement relationship by age or race. There was slight modification of the volunteering–illegal behavior relationship among the self-volunteers by sex in both waves 3 and 4, such that the rate ratio of self-volunteers vs non-volunteers among females was 0.81 (95 % CI: 0.69, 0.97), and that among males was 0.97 (95 % CI: 0.84, 1.1) in wave 3 (*p*-interaction = 0.0173), and the respective rate ratios were 0.58 (95 % CI: 0.42, 0.79) and 0.87 (95 % CI: 0.68, 1.1) in wave 4 (*p*-interaction = 0.0082).

The crude and adjusted wave 4 rate ratios and differences generated using generalized linear mixed models with only GSWGT134 weights (without our own stabilized inverse probability weights) generated very similar results to those reported in Table 2 and are hence not reported in the manuscript.

## Discussion

This is the first study to examine the long term association between adolescent volunteering (12–18 years age) and the incidence of illegal behaviors, arrests, and convictions in adulthood (>18 years age). The results suggest that those who self-volunteered reported 11 % fewer illegal behaviors, 31 % fewer arrests, and 39 % fewer convictions by age 18–28, and 28 % fewer illegal behaviors, 53 % fewer arrests, and 36 % fewer convictions by age 24–34, relative to the non-volunteers. The protective association of volunteering in our study is supported by an earlier short term follow up study (O’Donnell et al. 1999).

Adolescence is a formative period during which major moral and emotional development occurs. During this time, self-empowering experiences like volunteering may provide a sense of social responsibility, self-worth, and happiness, which helps in moral development. When these individuals grow they may become self-confident and responsible adults, who do not get involved in criminal activities. In other words, the protective association of adolescent volunteering with young adulthood criminal involvement may be mediated by enhanced sense of self-worth due to volunteering, which ultimately results in increased resilience, prosocial behavior, social responsibility, and greater happiness among youth (Batchelder and Root 1994; Giles and Eyler 1994; Reed et al. 2005; Raskoff and Sundeen 1999). Thus, volunteering may serve as a way for character development by empowering adolescents to become responsible adult members of the society.

Adult-required volunteering was also associated with fewer arrests and convictions as compared to not volunteering by wave 3, and fewer illegal behaviors, arrests, and convictions by wave 4. However, in wave 3 the adult-required volunteers had higher adulthood illegal behaviors as compared to non-volunteers. In addition, the overall association of adult-required adolescent volunteering with young adulthood criminal involvement was not as protective as that of self-volunteering. These differences could be due to differences in the type, quality, or intensity of volunteering experiences, including differences in the number of hours, volunteering activity, and level of engagement among the adult-required volunteers and self-volunteers. For example, religious groups, schools, or parents sometimes do annual or monthly volunteering retreats; conversely, self-volunteers may be motivated by specific causes and may be involved in them with great intensity as it may be a great source of self-worth and social interaction for them. In this study, we are not able to ascertain this potential qualitative difference in volunteering between adult-required and self volunteers due to the lack of such data. In addition, there may also be other social or environmental motivating factors in self-volunteers (over and above those controlled for in the analyses) that are not present in the adult-required volunteers, which may indicate other mechanisms of action. However, in this study and from the current literature we were not able to ascertain those additional mechanisms. Nevertheless, this association is suggestive of the potential protective effect of volunteering, especially when considered simultaneously with the self-volunteering—criminal involvement relationship.

The court-ordered volunteers had higher incidence of illegal behaviors, arrests, and convictions as compared to non-volunteers by wave 3 and wave 4. This is no surprise because court-ordered volunteering indicates prior crime involvement which is the strongest predictor of future involvement. (Resnick et al. 2004; Rowhani-Rahbar et al. 2015). These participants also had the highest mean delinquency score and were suspended and expelled from school most often than any other group at baseline. Simply put, non-volunteers are not the best comparison group for court-ordered volunteers. A better comparison group will be individuals who were convicted of the same crimes/ violations but asked to serve prison time/probation instead of volunteering or community service. This was examined in a study from Denmark where the author found that community service sentences were associated with lower violent crimes, higher incomes, and lower social services dependency after the completion of sentences (Andersen, 2015) and lower reconvictions (Klement, 2015), suggesting that these individuals became more responsible members of the society and contributed to the society.

The rates of crime involvement declined from wave 3 to wave 4 across all groups. This is analogous to that explained previously as the age-crime curve phenomenon where the crime rates increase until 18–25 years of age, after which they decline (Johnson et al. 2015; Moffitt 1993; Snyder, 2012; Sweeten et al. 2013). Volunteering, which is associated with greater social responsibility, self-worth, and happiness, may have a resilience building influence that accentuates the age-crime curve phenomenon resulting in more pronounced association in wave 4 than in wave 3.

The overall arrest rate for all participants between age 24–34 (wave-4) was 5.65 (95 % CI: 4.76, 6.53) per 100 person-years, which is an underestimate compared to the 2009 arrest rates presented by the Bureau of Justice Statistics (Bureau of Justice Statistics 2014). This may be because the Add Health wave 4 had very few 24, 25, and 32–34 year olds, due to which the wave 4 arrest rate does not represent all 24–34 year olds in 2009. The BJS statistics also involve all US youth between ages 24–34 in 2009, however, our rates represent only the school-enrolled youth during 1994–1995. Previous research suggests that youth attending school have less criminal involvement than youth not attending school (Lochner and Moretti 2004).

### Limitations

In this study, the baseline characteristics differed among the four groups of participants. Compared to the non-volunteers, self-volunteers, on average, had better social relationships, were raised in more economically stable families, had more educated and civically engaged parents, were more religious, were expelled or suspended from school less often, worked of pay more often, and also received allowance more often. This self-selection causes confounding and it may seem natural that these individuals will have better outcomes, even after controlling for all these factors, because there may be additional unknown confounders which we could not control for. However, it is notable that the adult-required volunteers were most privileged compared to the other three groups and yet, did not have as much protective association with the outcomes as self-volunteers. This may be because these volunteering subgroups may have qualitatively different volunteering experiences. However, we were not able to assess this because this information was not collected in the Add Health data. Similarly data on mediating factors were also not available hence we were not able to test the mediation of volunteering–criminal involvement relationship by mediators such as self-worth and feeling of social responsibility.

Schools have different volunteering requirement for their students. Some make it mandatory, while it is optional at other schools. Some schools coordinate such activities for students, while others don’t. These differences may change how school-based volunteering affects the outcomes, but in this study, all school based volunteering was combined into a single group with parent and religious groups based volunteering. This was a reflection of how data were collected in Add Health. Future studies should explore the effect of school-based volunteering programs on delinquency.

The exposure measure we used was not able to account for the level of engagement in volunteering, the type of volunteering activity, or the amount of time spent. These factors could modify the protective association observed in this study.

We did not have a count measure for the number of convictions in wave 4, hence we constructed a count variable using convictions from wave 3. The rates of convictions, thus obtained in wave 4, are underestimates of the true conviction rate for both exposed and unexposed. This may bias the wave 4 rate ratio and rate difference estimates. To address this, we conducted sensitivity analysis using the original ordinal outcome for convictions in wave 4. As compared to the non-volunteers, the adjusted odds ratio (OR) for conviction was 0.65 (95 % CI: 0.54, 0.78) for self-volunteers, 0.76 (95 % CI: 0.56, 1.0) for adult-required volunteers, and 2.0 (95 % CI: 1.5, 2.7) for court-ordered volunteers. The ORs approximate the adjusted RRs for wave 4 convictions (Table 2), suggesting that our rate ratio estimates are robust.

## Conclusions

Our study suggests that volunteering in adolescence may have a protective association with crime involvement in adulthood. Volunteering may have a potential of building long term resilience among adolescents. Future studies may evaluate the effectiveness of school-based volunteering programs, which already exist, in preventing criminal involvement over the life-course using prospective cohort or randomized trial designs. Such studies may also be well equipped to evaluate the effectiveness of the level of engagement, activity type, and time spent in volunteering.

## Additional file

Additional file 1: Directed acyclic graph (DAG) representing the association between adolescent volunteering and adulthood crime. (PNG 57 kb)

## Abbreviations

BJS: Bureau of Justice Statistics
CI: Confidence interval
DAG: Directed acyclic graph
FBI: Federal Bureau of Investigation
RR: Rate ratio
US DOJ: United States Department of Justice

## References

[CR1] Allen JP, Kuperminc G, Philliber S, Herre K (1994). Programmatic prevention of adolescent problem behaviors: The role of autonomy, relatedness, and volunteer service in Teen Outreach program. Am J Commun Psychol.

[CR2] Andersen SH (2015). Serving time or serving the community? Exploiting a policy reform to assess the causal effects of community service on income, social benefit dependency and recidivism. J Quant Criminol.

[CR3] Batchelder TH, Root S (1994). Effects of an undergraduate program to integrate academic learning and service: cognitive, prosocial cognitive, and identity outcomes. J Adolesc.

[CR4] Buckner JC, Mezzacappa E, Beardslee WR (2003). Characteristics of resilient youth living in poverty: The role of self-regulatory processes. Dev Psychopathol.

[CR5] Bureau of Justice Statistics (2014). Arrest Data Analysis Tool: Arrest rates by age in the US for all offenses, 1980-2012.

[CR6] Chen P, Chantala K (2014). Guidelines for analyzing Add Health Data.

[CR7] Crean HF (2012). Youth activity involvement, neighborhood adult support, individual decision making skills, and early adolescent behaviors: Testing a conceptual model. J Appl Dev Psychol.

[CR8] Erez A, Mikylincer M, van Ijzendoorn MH, Kroonenberg PM (2008). Attachment, personality, and volunteering: placing volunteerism in an attachment-theoretical framework. Pers Indiv Differ.

[CR9] Federal Bureau of Investigation (2013). Uniform Crime Reports: Crime trends, by metropolitan and nonmetropolitan counties by population group, 2012-2013 (Table 14).

[CR10] Gibson T (2008). Religion and civic engagement among America’s youth. Soc Sci J.

[CR11] Giles DE, Eyler J (1994). The impact of college community service laboratory on students’ personal, social, and cognitive outcomes. J Adolescence.

[CR12] Greenland S, Pearl J, Robins JM (1999). Causal diagram for epidemiologic research. Epidemiology.

[CR13] Greenland S, Daniel R, Pearce N (2016). Outcome modelling strategies in epidemiology: traditional methods and basic alternatives. Int J Epidemiol.

[CR14] Harris KM (2013). The Add Health Study: Design and Accomplishments.

[CR15] Herrenkohl TI, Maguin E, Hill KG (2000). Developmental risk factors for youth violence. J Adolesc Health.

[CR16] Hoffman JP, Xu J (2002). School activities, community service and delinquency. Crime Delinquency.

[CR17] Johnson WL, Giordano PC, Manning WD, Longmore MA (2015). The age-IPV curve: Changes in the perpetration of intimate partner violence during adolescence and young adulthood. J Youth Adolesc.

[CR18] Klement C (2015). Comparing the effects of community service and imprisonment on reconviction: results from a quasi-experimental Danish study. J Exp Criminol.

[CR19] Lansford JE, Dodge KA, Griffith Fontaine R, Bates JE, Pettit GS (2014). Peer rejection, affiliation with deviant peers, delinquency and risky sexual behavior. J Youth Adolesc.

[CR20] Lochner L, Moretti E (2004). The effect of education on crime: Evidence from prison inmates, arrests, and self-reports. Am Econ Rev.

[CR21] McKinney KG (2002). Engagement in community service among college students: is it affected by significant attachment relationships?. J Adolesc.

[CR22] Moffitt TE (1993). Adolescence-limited and life-course-persistent antisocial behavior: A developmental taxonomy. Psychol Rev.

[CR23] O’Donnell L, Stueve A, O’Donnell C (2002). Long-term reduction in sexual initiation and sexual activity among urban middle schoolers in the Reach for Health service learning program. J Adolesc Health.

[CR24] O’Donnell L, Stueve A, San Doval A (1999). Violence prevention and young adolescents’ participation in community service. J Adolesc Health.

[CR25] Raskoff SA, Sundeen RA (1999). Community service programs in high school. Law Contemp Probs.

[CR26] Reed VA, Christian Jernstedt C, Hawley JK, Reber ES, DuBois CA (2005). Effects of a small-scale, very short term service-learning experience on college students. J Adolesc.

[CR27] Resnick MD, Ireland M, Borowsky I (2004). Youth violence perpetration: What protects? What predicts? Findings from the National Longitudinal Study of Adolescent Health. J Adolesc Health.

[CR28] Rew L, Wong YJ (2006). A systematic review of associations among religiosity/ spirituality and adolescent health attitudes and behaviors. J Adolesc Health.

[CR29] Rowhani-Rahbar A, Zatzick D, Wang J (2015). Firearm-related hospitalization and risk for subsequent violent injury, death, or crime perpetration: A cohort study. Ann Intern Med.

[CR30] Sariaslan A, Långström N, D’Onofrio B (2013). The impact of neighborhood deprivation on adolescent violent criminality and substance misuse: A longitudinal, quasi-experimental study of the total Swedish population. Int J Epidemiol.

[CR31] Scharoun-Lee M, Adair LS, Kaufman JS, Gordon-Larsen P (2009). Obseity, race/ethnicity and the multiple dimensions of socioeconomic status during the transition to adulthood: A factor analysis approach. Soc Sci Med.

[CR32] Sweeten G, Piquero AR, Steinberg L (2013). Age and the explanation of crime, revisited. J Youth Adolesc.

[CR33] Snyder HN (2012). Arrests in the United States, 1990-2010.

[CR34] US Department of Justice (1999). Breaking the cycle of violence: Recommendations to improve criminal justice response to child victims and witnesses.

[CR35] Weuve J, Tchetgen Tchetgen EJ, Glymour MM (2012). Accounting for bias due to selective attrition: The example of smoking and cognitive decline. Epidemiology.

