# Supplementary figures and images for: Volunteering in adolescence and young adulthood crime involvement: a longitudinal analysis from the add health study
Source: Inj Epidemiol. 2016 Nov 21;3:26. doi: 10.1186/s40621-016-0091-6 (PMC5116440; doi:10.1186/s40621-016-0091-6)

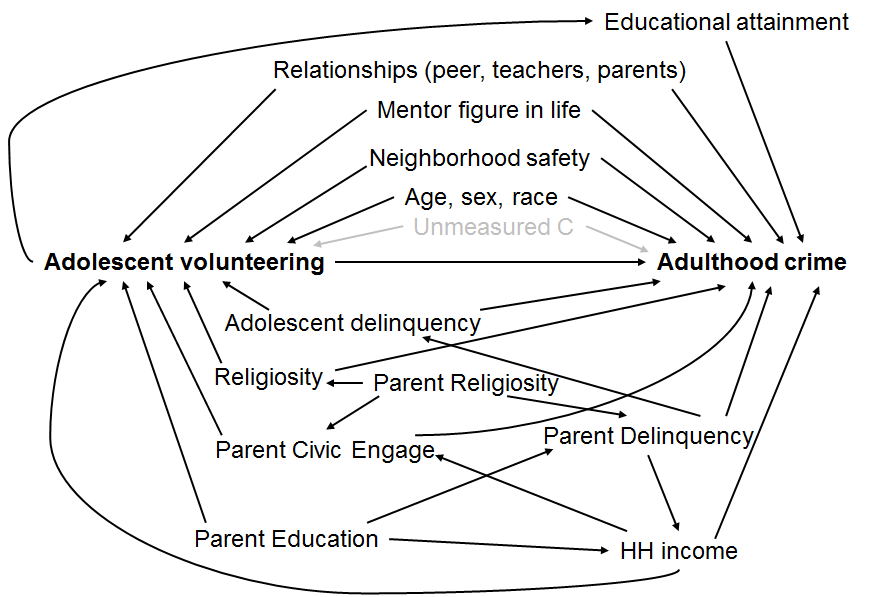

Supplement: Additional file 1: — Directed acyclic graph (DAG) representing the association between adolescent volunteering and adulthood crime. (PNG 57 kb) [file 40621_2016_91_MOESM1_ESM.png]
